# Disaster Response Team FAST Skills Training with a Portable Ultrasound Simulator Compared to Traditional Training: Pilot Study

**DOI:** 10.5811/westjem.2015.1.23720

**Published:** 2015-03-06

**Authors:** Michael T. Paddock, John Bailitz, Russ Horowitz, Basem Khishfe, Karen Cosby, Michelle J. Sergel

**Affiliations:** John H. Stroger, Jr. Hospital of Cook County, Department of Emergency Medicine, Chicago, Illinois

## Abstract

**Introduction:**

Pre-hospital focused assessment with sonography in trauma (FAST) has been effectively used to improve patient care in multiple mass casualty events throughout the world. Although requisite FAST knowledge may now be learned remotely by disaster response team members, traditional live instructor and model hands-on FAST skills training remains logistically challenging. The objective of this pilot study was to compare the effectiveness of a novel portable ultrasound (US) simulator with traditional FAST skills training for a deployed mixed provider disaster response team.

**Methods:**

We randomized participants into one of three training groups stratified by provider role: Group A. Traditional Skills Training, Group B. US Simulator Skills Training, and Group C. Traditional Skills Training Plus US Simulator Skills Training. After skills training, we measured participants’ FAST image acquisition and interpretation skills using a standardized direct observation tool (SDOT) with healthy models and review of FAST patient images. Pre- and post-course US and FAST knowledge were also assessed using a previously validated multiple-choice evaluation. We used the ANOVA procedure to determine the statistical significance of differences between the means of each group’s skills scores. Paired sample t-tests were used to determine the statistical significance of pre- and post-course mean knowledge scores within groups.

**Results:**

We enrolled 36 participants, 12 randomized to each training group. Randomization resulted in similar distribution of participants between training groups with respect to provider role, age, sex, and prior US training. For the FAST SDOT image acquisition and interpretation mean skills scores, there was no statistically significant difference between training groups. For US and FAST mean knowledge scores, there was a statistically significant improvement between pre- and post-course scores within each group, but again there was not a statistically significant difference between training groups.

**Conclusion:**

This pilot study of a deployed mixed-provider disaster response team suggests that a novel portable US simulator may provide equivalent skills training in comparison to traditional live instructor and model training. Further studies with a larger sample size and other measures of short- and long-term clinical performance are warranted.

## INTRODUCTION

Pre-hospital focused assessment with sonography in trauma (FAST) has been reported to be accurate and effective for triage and early diagnosis during numerous mass casualty events throughout the world.^1^–^9^ As with any clinical task, proper knowledge and skills training is essential to ensure appropriate healthcare provider utilization and optimal clinical outcomes. Traditional FAST training requires didactic training and on-site expert live instructor skills training using healthy models with significant logistical challenges and cost.

Recently a portable ultrasound (US) simulator, the SonoSim laptop training solution, was developed specifically to rapidly train healthcare providers in the knowledge and skills of FAST. Review of narrated screencasts teaches the required knowledge. Deliberate practice obtaining patient US images with the SonoSim gyrometer probe and real-time simulator feedback teaches the necessary hands-on skills of image acquisition and interpretation.

A recent systematic review of 14 US simulation training studies reported a wide variability in research design and called for further investigation prior to widespread use.^10^ Most studies have used medical students and other novice ultrasonographers. However, no study has described the effectiveness of a portable US simulator for skills training in comparison to traditional skills training for a mixed provider disaster response team.

The primary objective of this pilot study was to describe the effectiveness of novel portable US simulators in teaching the skills of the FAST examination in comparison to traditional training for a deployed mixed-provider disaster response team.

## METHODS

We conducted a prospective randomized blinded trial of SonoSim versus traditional FAST skills training. The study was conducted at a satellite institution, Provident Hospital of Cook County, during the May 2012 Chicago North Atlantic Treaty Organization (NATO) Summit. Disaster response team members from Colorado and California were stationed at the hospital in the event of a crisis situation. As part of standard deployment training, a four-hour FAST US course was conducted by the Department of Emergency Medicine, Division of Emergency US, John H. Stroger, Jr. Hospital of Cook County.

The initial idea for this pilot investigation, study objective, methodology, and analysis were developed and conducted in collaboration between the Division of Emergency US and the Division of Emergency US and the Cook County Simulation program without industry involvement. US equipment (Sonosite TITAN, Bothell, WA) for the course was provided by Sonosite, Inc. Portable US simulators (SonoSim Ultrasound Training Solution, Point of Care Edition, Santa Monica, CA) were provided for the course by SonoSim, Inc.. No financial support was provided. Local institutional review acknowledgment was obtained for this exempt educational study of de-identified trainees.

The combined Colorado and California disaster response team was comprised of 36 mixed provider team members. Participation in this research project was strictly voluntary. After discussion of the educational course and research project, all disaster response team members agreed to participate and signed an informed consent.

At the start of the course, all participants first completed a pre-course US and FAST knowledge test previously developed by experts at the University of California at Irvine.^11^ The 74-item multiple-choice test was validated in an earlier medical student examination of SonoSim by the Center for Research on Evaluation, Standards, and Student Testing.^11^ The test assessed key content contained in SonoSim’s narrated screencast didactics; specifically knowledge of anatomy, indications, contraindications, image acquisition, image interpretation, and clinical integration of US findings.

To learn the required FAST knowledge, all participants then reviewed US simulator-based narrated screencast didactics covering US physics, machine use, and FAST for 90 minutes. These didactics were created by national experts consulting with SonoSim, and reviewed by local investigators independently to ensure quality and absence of industry bias. No skills training was provided during this knowledge learning session.

**Figure 1:**
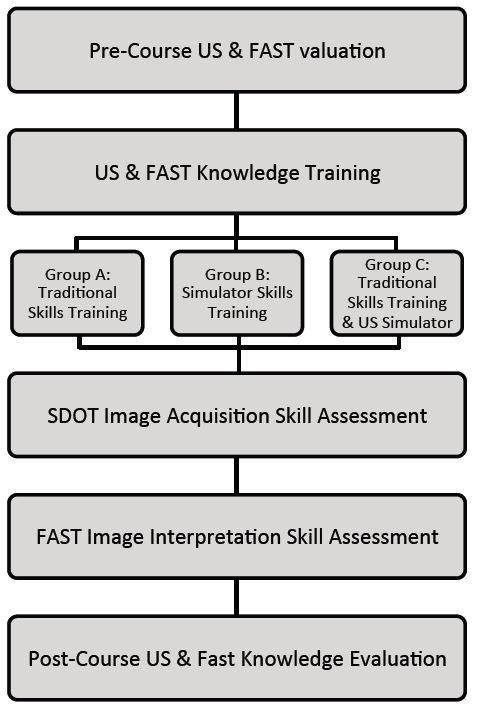
Study design. *US,* ultrasound; *FAST*, focused assessment of sonography in trauma; *SDOT*, standardized direct observation

Next, disaster response team members were stratified by provider role into physicians, nurses, paramedics, and other clinicians. The participants were then randomly assigned to one of three training groups: Group A. Traditional Skills Training, Group B. US Simulator Skills Training and Group C: Traditional Skills Trainings plus US simulator Skills Training. See Figure 1 for course and research study flowchart. For each participant we recorded basic demographic information including age, gender, provider role (physician, nurse, or paramedic), and prior US experience.

To learn the required FAST skills, participants then completed skills training based on group randomization. Each group was divided into three subgroups to create a 1:4 instructor/simulator to participant ratio. Skills training sessions lasted one hour during which all four participants in the subgroups received approximately 15 minutes of hands-on time. Group A and B received one hour of skills training while Group C received two hours of skills training.

After the skills training, to measure post-intervention skill in image acquisition, all participants individually completed a FAST standardized direct observation tool (SDOT) on a live healthy model. Evaluators were blinded to participant’s provider role and prior US experience. Participants were asked to obtain each of the four FAST views and press freeze in less than one minute per view. Images were rated by the evaluator on a 1–5 scale (1 – no useful information provided by the image, 3 – adequate image quality and visualization of relevant anatomy to make a clinical decision, 5 – textbook quality image). Evaluators were all US faculty, US fellows, or senior emergency medicine (EM) residents with US case totals above the American College of Emergency Physician’s minimum benchmark of 150 emergency US exams. For the research project, evaluators were specifically given detailed instructions and provided with examples of all FIVE ratings in each FAST view during a two-hour faculty-only walk through session prior to the course.

To measure post-training skills in image interpretation, all participants individually completed a FAST image interpretation assessment comprised of a review of FAST images from four actual trauma cases. Participants identified the anatomical location of the obtained image as well as whether the image was normal or revealed free fluid. Lastly, all participants completed the same post-course US and FAST knowledge test.

Primary endpoints included FAST image acquisition and interpretation skills assessment scores. Secondary endpoints included pre-course and post-course US and FAST knowledge test scores.

The research project, including the administration and collection of study related forms, was completed by a team of four volunteer research assistants under the supervision of an experienced clinical research coordinator (EC). Two emergency US fellowship directors (JB and KC), coordinated logistics of the educational course and ensured that all participants benefitted from the learning experience.

We used the ANOVA procedure to determine the significance of group differences in FAST image acquisition and interpretation skills assessment scores. Two-tailed paired t-tests were conducted for each group to assess pre-course and post-course differences on the US and FAST knowledge test scores. We also used the ANOVA procedure to determine if any significant differences existed among groups on the knowledge test scores. We performed statistical analysis using SPSS version 21.0 (IBM Corp., Armonk, NY).

## RESULTS

All 36 disaster response team members participated in the course and research project. The demographic data and baseline characteristics of study participants are summarized in Table. In each group, four of the twelve participants had received prior US training.

**Figure 2:**
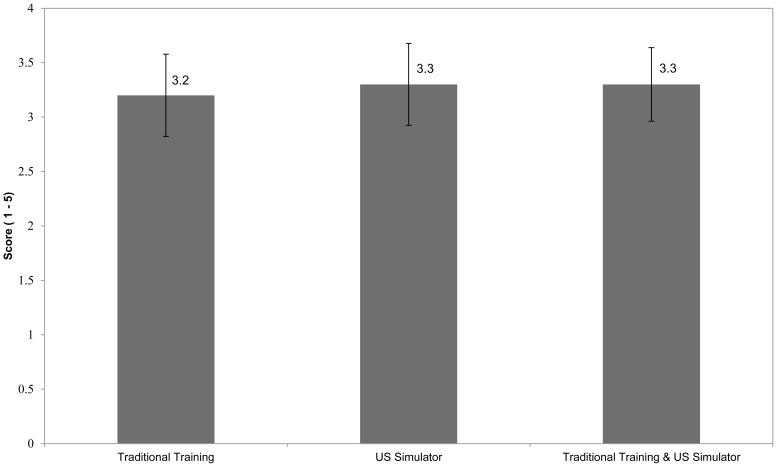
SDOT image acquisition scores across groups. *SDOT*, standardized direct observation tool; *US*, ultrasound Data are reported as mean with 95% CI. Images were rated by the evaluator on a 1–5 scale (1 – No information provided by the image, 3 – Adequate image to make a clinical decision, 5 – Textbook quality image).

The primary endpoints of FAST image acquisition assessment scores between training groups are depicted in Figure 2. In each group, the mean image acquisition skills score was above 3 - acceptable to make a clinical decision. An additional ordinal and dependent analysis of image acquisition scores using a Krukal-Wallis test confirmed similarity between all groups. Likewise, mean image interpretation skills score was similar across groups. The mean (± SD) image interpretation score by group was; Group A. 76 ± 6%, Group B. 77 ± 6%, and Group C. 81 ± 6%. The ANOVA procedure did not demonstrate any statistically significant differences between the training groups’ FAST image acquisition or image interpretation skills scores.

**Figure 3:**
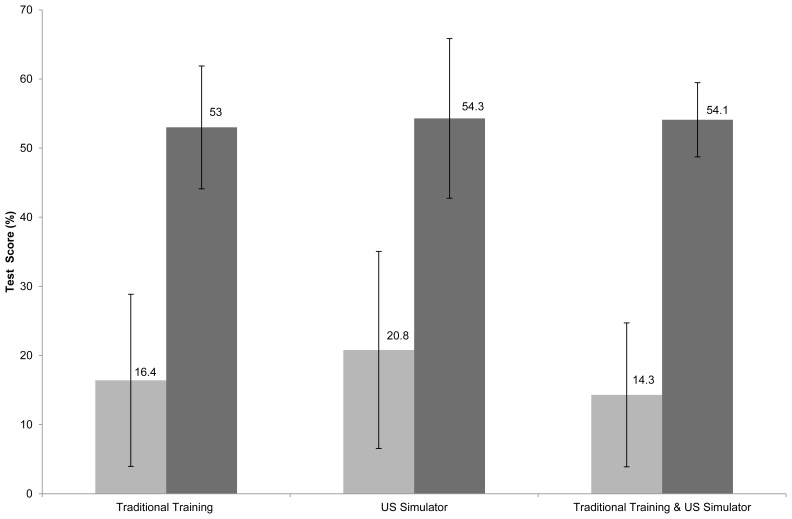
US and FAST knowledge pre- and post-evaluation scores. *US*, ultrasound; *FAST*, focused assessment of sonography in trauma Data are reported as mean with 95% CI. Lighter grey designates pre-course examination scores, darker grey designated post-course examination.

The secondary endpoint of pre- and post-course US and FAST knowledge test scores are shown in Figure 3. Within each training group, there was a statistically significant improvement in the mean pre- and post-course knowledge test scores (p<0.001 in each group). The ANOVA procedure again did not reveal any significant differences between the three training groups’ pre- and post-course knowledge gains (p>0.05).

## DISCUSSION

FAST has been demonstrated to be an invaluable primary triage and diagnostic tool in multiple disasters even in remote locations.^1^–^6^,^9^ Effective utilization of FAST by disaster response teams requires proper training. However, FAST training for disaster response team members has numerous logistical challenges. Although FAST knowledge may be effectively learned with narrated didactics, traditional skills training requires numerous US machines, instructors, models, and time.^12^,^13^ Fortunately, over the last decade US simulators have been developed to quickly teach these skills.

Our results are similar to earlier work describing the utility of US simulators.^10^ In a prospective randomized controlled trial, Girzadas et al. reported improvements in both EM resident learning and the evaluation using a pelvic US simulator.^14^ Likewise, in a prospective study, Lee et al. demonstrated improved EM resident performance and confidence with a central venous insertion US simulator.^15^ Outside of EM, Burden et al. reported that both novices and experts were able to adequately obtain images and specific measurements in a prospective cross-sectional comparative study utilizing an obstetric US simulator.^16^ Similarly, Platts et al. demonstrated effective use of computer-based transthoracic and transesophageal echocardiogram simulators in acquiring images.^17^

Our pilot study is the first to compare traditional FAST skills training to a novel portable US simulator for members of a deployed disaster response team. Training with the US simulator appears to result in equivalent disaster response team member skill performance in both image acquisition and interpretation when compared to traditional FAST skills training. Furthermore, the provision of both types of training did not result in a statistically significant improvement over either alone, suggesting that the US simulator alone may be sufficient for FAST skills training.

Additionally, these initial results suggest that FAST skills may be easily taught to disaster response team members with non-physician healthcare providers. Similarly, Heegaard et al. reported that paramedics undergoing two training sessions were able to adequately perform and interpret pre-hospital FAST and abdominal aorta US exams.^4^ This cross-role training and familiarity with performing and interpreting FAST may be essential in mass casualty events.

## LIMITATIONS

Our pilot study has several limitations. The sample size was fixed by the number of disaster response team members deployed at Provident Hospital of Cook County during the 2012 NATO Summit. The majority of the evaluators were emergency attending US faculty or fellows. EM residents conducted two of the six evaluation stations. These two EM residents were PGY4 resident members of our EM US Resident College (a resident scholarly tract), each with over 300 total exams completed during residency that was finishing the next month (June 2012). To ensure examiner reliability, a course and study walk through was conducted the day before for all evaluators and repeated the morning of the course. Scores did not appear to vary significantly between evaluation stations (unpublished data). Although we evaluated pre- and post-course US and FAST knowledge only post-course FAST skills were evaluated due to educational course logistics. However, most trainees had no prior US training prior to this course. Furthermore, the lengthy US and FAST knowledge test was designed specifically for medical student learners. This resulted in lower than expected pre- and post-course scores among our mixed providers but similar overall improvement versus prior studies.^11^ Future larger prospective, randomized, blinded studies will continue to compare different learning modalities for rapidly developing short and long term US knowledge and skills among disaster response team members.

## CONCLUSION

For deployed mixed provider disaster response team members with limited training time and resources, a portable US simulator appears to provide equivalent skills training in comparison to traditional live instructor and model training in this initial pilot study. Future work will focus on evaluating larger teams of mixed providers as well as long and short-term clinical performance of FAST skills.

## Figures and Tables

**Table:** Baseline characteristics of participants in FAST training.

	Traditional training n=12	US simulator n=12	Traditional training + US simulator n=12
Mean age (years)	50 ± 11	43 ± 13	43 ± 9
Sex			
Male	7 (58)	10 (83)	7 (58)
Female	5 (42)	2 (17)	5 (42)
Occupation			
Nurse	3 (25)	3 (25)	3 (25)
Physician	5 (42)	5 (42)	4 (33)
Paramedic/EMT	4 (33)	4 (33)	5 (42)

Data are reported as n (%) or mean ± SD. No significant differences between groups (p>0.05).

*FAST*, focused assessment with sonography in trauma; *US*, ultrasound; *EMT*, emergency medical technician
